# Identification of genes associated with cancer progression and prognosis in lung adenocarcinoma: Analyses based on microarray from Oncomine and The Cancer Genome Atlas databases

**DOI:** 10.1002/mgg3.528

**Published:** 2018-12-16

**Authors:** Wei Liu, Songyun Ouyang, Zhigang Zhou, Meng Wang, Tingting Wang, Yu Qi, Chunling Zhao, Kuisheng Chen, Liping Dai

**Affiliations:** ^1^ Department of Gastroenterology in the First Affiliated Hospital Zhengzhou University Zhengzhou China; ^2^ Department of Respiratory and Sleep Medicine in the First Affiliated Hospital Zhengzhou University Zhengzhou China; ^3^ Department of Radiology in the First Affiliated Hospital Zhengzhou University Zhengzhou China; ^4^ Department of Medical Examination in the First Affiliated Hospital Zhengzhou University Zhengzhou China; ^5^ Department of Thoracic Surgery in the First Affiliated Hospital Zhengzhou University Zhengzhou China; ^6^ Department of Pathology in the First Affiliated Hospital Zhengzhou University Zhengzhou China; ^7^ Department of Tumor Research in the Institute of Medical and Pharmaceutical Sciences Zhengzhou University Zhengzhou China

**Keywords:** biomarker, lung adenocarcinoma, microarray, prognosis

## Abstract

**Background:**

Lung adenocarcinoma (LUAD) accounts for approximately 40% of all lung cancer patients. There is an urgent need to understand the mechanisms of cancer progression in LUAD and to identify useful biomarkers to predict prognosis.

**Methods:**

In this study, Oncomine database was used to identify potential genes contributed to cancer progression. Bioinformatics analysis including pathway enrichment and text mining was used to explain the potential roles of identified genes in LUAD. The Cancer Genome Atlas database was used to analyze the association of gene expression with survival result.

**Results:**

Our results indicated that 80 genes were significantly dysregulated in LUAD according to four microarrays covering 356 cases of LUAD and 164 cases of normal lung tissues. Twenty genes were consistently and stably dysregulated by more than twofold. Ten of 20 genes had a relationship with overall survival or disease‐free survival in a cohort of 516 LUAD patients, and 19 genes were associated with tumor stage, gender, age, lymph node, or smoking. Low expression of *AGER* and high expression of *CCNB1* were specifically associated with poor survival.

**Conclusion:**

Our findings implicate *AGER* and *CCNB1* might be potential biomarkers for diagnosis and prognosis targets for LUAD.

**Clinical practice points**

1. An increasing number of studies used public databases, such as Oncomine and The Cancer Genome Atlas (TCGA), as powerful evidence to screen and identify novel biomarkers for diagnosis and prognosis.
2. In the present study, using mRNA expression profiles retrieved from Oncomine online database, we identified 80 dysregulated genes in LUAD and annotated several biological processes closely associated with the progression and development of LUAD. For the 20 stably and consistently dysregulated genes in LUAD, we retrieved the data of mRNA expression, clinical information from TCGA LUAD project to identify genes associated with cancer prognosis in LUAD.
3. The findings indicate that *AGER* and *CCNB1* might be useful biomarkers for diagnosis and prognosis and could be potential therapeutic targets for LUAD treatment in the clinical work.

## 1 INTRODUCTION

Lung cancer (LC), especially non‐small‐cell lung cancer (NSCLC), is the leading cause of cancer death worldwide and is associated with significant morbidity and poor prognosis (Mehta, Dobersch, Romero‐Olmedo, & Barreto, 2015; Siegel, Naishadham, & Jemal, 2013). Lung adenocarcinoma (LUAD), a subtype of NSCLC, accounts for approximately 40% of all lung cancer patients and is one of the most genetically characterized human epithelial malignancies (Bender, 2014). In spite of recent improvement in clinical therapy, 5‐year survival rate of NSCLC patients remains lower than 20% (Allemani et al., 2015), due to the low diagnosis rate at early stage and the frequent cancer recurrence and metastasis. There is an urgent need to identify novel diagnostic and prognostic markers to improve the survival of LC patients.

Bioinformatics analyses, including usage of microarray expression datasets (Stuart, Segal, Koller, & Kim, 2003), protein/gene–protein/gene interaction networks (Ivanov et al., 2018), and the annotation of genes (Phuong & Nhung, 2013), are being utilized as a powerful tool to study the cancer progression and to identify serum biomarkers (Hormigo et al., 2006; Huddleston, Wong, Welch, Berkowitz, & Mok, 2005) as well as potential therapeutic targets (Armstrong et al., 2003; Ye et al., 2003). Large amounts of data generated by this tool are collected in public archives such as the major public projects The Cancer Genome Atlas (TCGA) (DeSantis, Ma, Bryan, & Jemal, 2014), Oncomine (Rhodes et al., 2004), Gene Expression Omnibus (GEO) (Barrett et al., 2013), and so on. An increasing number of studies used these public databases as powerful evidence to screen and identify novel biomarkers for diagnosis and prognosis. For instance, by retrieving data from Oncomine and TCGA, Yin et al. (2016) successfully identified a group of genes related to cancer progression and prognosis in hepatocellular carcinoma. Liu et al. (2015) identified six genes that may be potential therapeutic targets and biomarkers for diagnosis and prognosis in ovarian cancer, based on data retrieved from Oncomine, GEO, and TCGA. Thus, bioinformatics analysis is a feasible and valuable method to mine data and predict gene function.

In this study, using mRNA expression profiles retrieved from Oncomine online database, we identified 80 dysregulated genes in LUAD and annotated several biological processes closely associated with the progression and development of LUAD. For the 20 stably and consistently dysregulated genes in LUAD, we retrieved the data of mRNA expression, clinical information from TCGA LUAD project to identify genes associated with cancer prognosis in LUAD.

## 2 MATERIALS AND METHODS

### 2.1 Data source

Microarrays data were selected from Oncomine database (http://www.oncomine.org/resource/login.html). Initially, 12 datasets were found when we used the following filters: (a) analysis type: differential analysis—cancer versus normal analysis; (b) cancer type: lung cancer—non‐small‐cell lung cancer; and (c) dataset filters: data type—mRNA. In order to retrieve the stably and consistently dysregulated genes in LUAD, we subsequently selected four studies from the 12 datasets according to the criteria: (a) lung adenocarcinoma versus normal; (b) sample number more than 50; and (c) microarray platform is Human Genome U133 or U133 Plus 2.0. Finally, genes that were significantly dysregulated in LUAD tissues were identified based on four microarrays studies: Hou Lung (45 LUADs vs. 65 lung tissues) (Hou et al., 2010), Landi Lung (58 LUADs vs. 49 lung tissues) (Landi et al., 2008), Okayama Lung (226 LUADs vs. 20 lung tissues) (Okayama et al., 2012), and Su Lung (27 LUADs vs. 30 lung tissues) (Su et al., 2007). The four studies totally include 356 cases of LUAD and 164 cases of normal lung tissues (Supporting Information Table S1). The rank for a gene is the median rank for that gene across each of the analysis. mRNA expression and clinical information, including age, gender, smoking status, overall survival time (OS), disease‐free survival time (DFS), TNM stage, metastasis, and lymph node metastasis, of 522 LUAD patients in a TCGA cohort were retrieved from TCGA database (https://cancergenome.nih.gov/), but only 516 samples with matched gene expression data and clinical data were utilized to analyze the clinical importance of the genes identified in this study.

### 2.2 Bioinformatics analyses

Gene Ontology (GO) term supplies the annotation of genes and describes functions of genes or their proteins from three categories: cellular component (CC), biological process (BP), and molecular function (MF) (Gene Oncology Consotorium, 2015; Harris et al., 2004). The Kyoto Encyclopedia of Genes and Genomes (KEGG, http://www.genome.jp/) is a widely used database that supplies the molecular functions of genes and proteins (Kanehisa, Sato, Kawashima, Furumichi, & Tanabe, 2016). The Database for Annotation, Visualization, and Integrated Discovery (DAVID, Jiao et al., 2012) contains a comprehensive biological knowledge and a series of analytic tools available for extracting biological themes for genes or proteins. GO enrichment analysis and KEGG pathway enrichment analysis of target genes were performed using the DAVID online tool. The *p*‐value <0.05 was chosen as the cutoff criterion for both GO functional enrichment analysis and KEGG pathway. Function prediction based on text mining was performed using the Coremine Medical online database (http://www.coremine.com/medical/).

### 2.3 Data analysis

Expression values of gene were categorized as high and low expression using the median value as a cutoff for clinical characteristics in a TCGA cohort. The association of gene expression frequency with age, gender, smoking status, TNM stages, and lymph node numbers was analyzed by Pearson’s chi‐square test. The Kaplan–Meier method and log‐rank test were used for survival analyses. Univariate and multivariate Cox proportional hazards regression models were used to calculate a hazard ratio (HR) for overall survival (OS) and disease‐free survival (DFS) according to the gene expression status (high or low). Statistical analyses were performed at the two‐tailed α level of 0.05, using SPSS software version 21.0.

## 3 RESULTS

### 3.1 Retrieval of significantly dysregulated genes in LUAD

**Figure 1:**
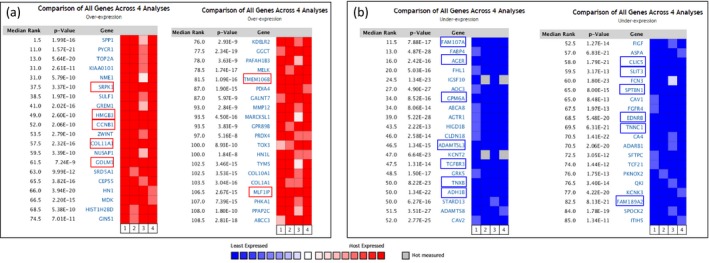
The 80 genes significantly dysregulated in lung adenocarcinoma (LUAD) according to four independent microarrays retrieved from the Oncomine database. (a) The top 40 genes upregulated in microarrays. (b) The top 40 genes downregulated in microarrays. The four microarrays cover a total of 356 cases of LUAD and 164 cases of normal lung tissues. The rank for a gene is the median rank for the gene across each of the analysis. The *p* value given for a gene is for the median‐ranked analysis. Genes with red and blue box were significant and consistent overexpression and underexpression in the four studies

Four independent microarray datasets deposited in the Oncomine database were selected to identify genes associated with LUAD development and progression (Supporting Information Table S1). Based on comparison of all genes across four datasets performed by Oncomine online tool, 40 genes significantly upregulated (*p* < 5.16E−8) and 40 genes significantly downregulated (*p* < 1.34E−11) in LUAD were retrieved (Figure 1).

**Figure 2:**
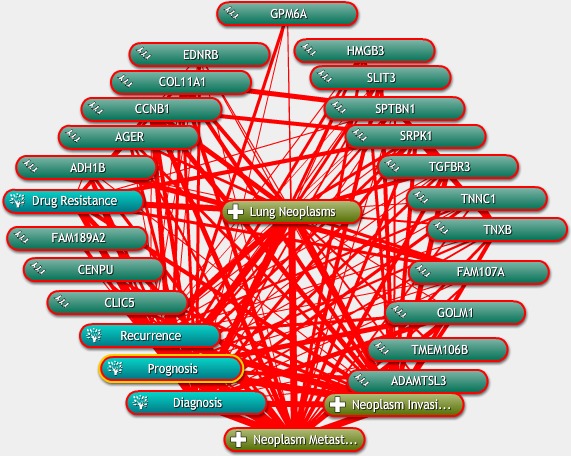
Association of the genes with LUAD characteristics was determined by text mining using Coremine Medical and probabilistic scoring (*p* < 0.05)

Among the 80 genes that were dysregulated in LUAD according to four independent microarray datasets covering a total of 356 cases of LUAD and 164 cases of normal lung tissues, seven genes (*SRPK1*,* HMGB3*,* CCNB1*,* COL11A1*,* GOLM1*,* TMEM106B*, and *MLF1IP*) were stably and consistently upregulated and 13 genes (*FAM107A*,* AGER*,* GPM6A*,* ADAMTSL3*,* TGFBR3*,* TNXB*,* ADH1B*,* CLIC5*,* SLIT3*,* SPTBN1*,* EDNRB*,* TNNC1*, and *FAM189A2*) were stably and consistently downregulated in LUAD (Figure 2; Supporting Information Table S2).

### 3.2 Functional enrichment analyses

Gene Ontology functional enrichment analysis of these 80 genes using the DAVID online tool indicated that total 33 terms were significantly enriched (Supporting Information Table S3). Extracellular matrix organization and response to drug were the top two biological processes, covering 12 genes, extracellular region was the top cellular component, covering 14 genes, and protein binding was the top molecular function, covering 45 genes (Supporting Information Table S3). The results of KEGG pathway enrichment analysis showed that the 80 genes were only enriched in pathway of focal adhesion (Supporting Information Table S3).

### 3.3 Potential roles of the genes in LUAD progression

The potential roles of the 20 genes in LUAD were predicted on the basis of Coremine Medical mining. As shown in Figure 2, the associations of the genes with diagnosis, prognosis, drug resistance, recurrence, metastasis, and invasiveness of LUAD were comprehensively analyzed. The results indicated that the 20 genes were all associated with at least one factor contributing to cancer progression, and many of the genes, for example, *MLF1IP*,* SRPK1*,* CCNB1*,* COL11A1*,* ADH1B*,* SPTBN1,* and *EDNRB,* were closely associated with all of the factors included in this analysis. Nineteen genes were associated with diagnosis with the exception of *MLF1IP*. Eighteen genes were associated with metastasis except for *ADAMTSL3* and *FAM189A2*. With the exception of *TMEM106B* and *FAM189A2*, the other 18 genes were associated with prognosis. Most of the genes were extensively associated with several factors. For instant, *AGER* was associated with invasiveness, metastasis, diagnosis, and prognosis, and *CCNB1* was associated with invasiveness, metastasis, diagnosis, prognosis, drug resistance, and recurrence (Figure 2).

### 3.4 Analysis of clinical magnitude

The clinical magnitude of the 20 stably and consistently dysregulated genes in LUAD was assessed on the basis of TCGA clinical data. A total of 522 patient samples with LUAD were retrieved from a cohort of TCGA database, while only 516 samples with mRNA expression value were available to analyze the association of gene expression with clinical characteristics. The gene expression level was categorized as high or low based on the median value referring to a previous study (Yin et al., 2016).

The association of gene expression with tumor stage, lymph node, metastasis, age, gender, and smoking pack‐year was analyzed. Eight genes were significantly associated with stage (*p* < 0.05; Table 1), in which high expression of *ADH1B*,* AGER*,* CLIC5*,* FAM107A,* and *GPM6A* was associated with early stage, while *CCNB1*,* CENPU,* and *GOLM1* was associated with late stage of LUAD. Especially, *CCNB1* and *CENPU* were markedly and highly expressed in stage III (63.1%, *p* = 0.001) and IV (60.0%, *p* = 0.040), respectively. Seven genes had a relationship with lymph node, where the frequency of high expression of *CCNB1*,* CENPU*,* COL11A1,* and *TMEM106B* was significantly higher in patients with more than one lymph node than that without one (Table 1). We observed that four genes (*ADH1B*,* FAM107A*,* SLIT3,* and *TNXB*) were highly expressed in female patients, while other four genes (*CCNB1*,* CENPU*,* HMGB3,* and *SRPK1*) were highly expressed in male patients. In addition, three genes (*ADH1B*,* CLIC5,* and *GPM6A*) were expressed at high levels in LUAD patients aged ≥65 years, while two genes (*CCNB1* and *CENPU*) were expressed at high level in patients aged <65 years. Finally, six genes were closely related to the smoking status. Two genes (*CENPU* and *TMEM106B*) showed high expression in patients with smoking ≥40 pack‐year, the other four genes (*ADAMSTL*,* EDNRB*,* FAM107A,* and *TGFBR3*) showed high expression in patients with smoking <40 pack‐year (Table 1).

**Table 1:** Association of gene expression (high level) with tumor stage, lymph node, age, gender, and smoking in 516 patients with lung adenocarcinoma (LUAD)

Stage	*N* = 508	*ADH1B*	*AGER*	*CCNB1*	*CENPU*	*CLIC5*	*FAM189A2*	*GOLM1*	*GPM6A*
I	277	157 (56.7)	154 (55.6)	117 (47.2)	123 (44.4)	159 (57.4)	159 (57.4)	126 (45.5)	153 (55.2)
II	122	52 (42.6)	53 (43.4)	71 (58.2)	70 (57.4)	56 (45.9)	53 (43.4)	69 (56.6)	56 (45.9)
III	84	31 (36.9)	34 (40.5)	53 (63.1)	47 (56.0)	29 (34.5)	33 (39.3)	50 (59.5)	31 (36.9)
IV	25	12 (48.0)	12 (48.0)	14 (56.0)	15 (60.0)	9 (36.0)	8 (32.0)	10 (40.0)	12 (48.0)
	*p*	0.004	0.034	0.001	0.040	0.001	0.002	0.039	0.022
**Lymph node**	***N* = 515**	***ADH1B***	***CCNB1***	***CENPU***	***CLIC5***	***COL11A1***	***FAM189A2***	***TMEM106B***	
0	333	181 (54.4)	148 (44.4)	155 (46.5)	182 (54.7)	155 (46.5)	182 (54.7)	150 (45.0)	
≥1	182	76 (41.8)	110 (60.4)	103 (56.6)	75 (41.2)	104 (57.1)	75 (41.2)	108 (59.3)	
	*p*	0.006	0.001	0.029	0.004	0.022	0.004	0.002	
**Gender**	***N* = 516**	***ADH1B***	***CCNB1***	***CENPU***	***FAM107A***	***HMGB3***	***SLIT3***	***SRPK1***	***TNXB***
Male	239	107 (44.8)	135 (56.5)	134 (56.1)	104 (43.5)	136 (56.9)	108 (45.2)	135 (56.5)	101 (42.3)
Female	277	151 (54.5)	123 (44.4)	124 (44.8)	154 (55.6)	123 (44.4)	151 (54.5)	124 (44.8)	157 (56.7)
	*p*	0.027	0.006	0.010	0.006	0.005	0.035	0.008	0.001
**Age**	*N* = 497	***ADH1B***	***CCNB1***	***CENPU***	***CLIC5***	***GPM6A***			
<65	221	94 (42.5)	123 (55.7)	125 (56.6)	86 (38.9)	95 (43.0)			
≥65	276	155 (56.2)	122 (44.2)	120 (43.5)	159 (57.6)	151 (54.7)			
	*p*	0.003	0.011	0.004	0.000	0.009			
**Smoking**	***N* = 349**	***ADAMSTL***	***CENPU***	***EDNRB***	***FAM107A***	***TGFBR3***	***TMEM106B***		
<40 pack‐year	172	93 (54.1)	77 (44.8)	98 (57.0)	98 (57.0)	88 (51.2)	65 (37.8)		
≥40 pack‐year	177	76 (42.9)	107 (60.5)	76 (42.9)	81 (45.8)	69 (39.0)	92 (52.0)		
	*p*	0.037	0.003	0.009	0.036	0.022	0.008		

Note

*p*: chi‐square test.

### 3.5 Survival analysis of 20 genes

**Figure 3:**
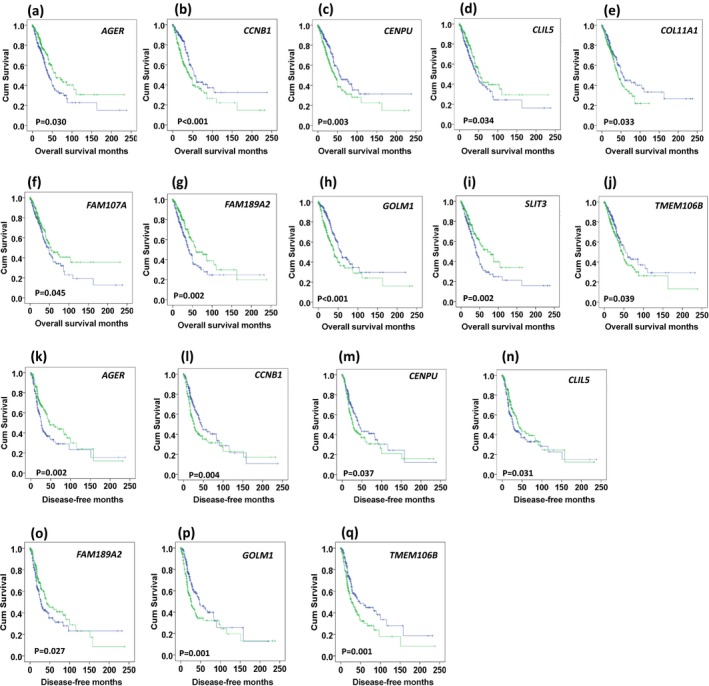
Association of genes with OS or DFS, analyzed by Kaplan–Meier survival plots. (a–j) Association of ten genes (*AGER, CCNB1, CENPU, CLIL5, COL11A1, FAM107A, FAM189A2, GOLM1, SLIT3, and TMEM106B*) with OS. (k–q) Association of seven genes (*AGER, CCNB1, CENPU, CLIL5, FAM189A2, GOLM1, and TMEM106B*) with DFS. Expression levels of a gene were dichotomized into high expression (green line) and low expression (blue line) using the median as a threshold. DFS: disease‐free survival; OS: overall survival

Ten out of 20 genes had a relationship with OS and/or DFS (Supporting Information Table S4). Seven genes were associated with both OS and DFS. Low expression of *AGER* (Figure 3a,k), CLIC5 (Figure 3d,n), and *FAM189A2* (Figure 3g,o), and high expression of *CCNB1* (Figure 3b,l), *CENPU* (Figure 3c,m), *GOLM1* (Figure 3h,p), and *TEME106B* (Figure 3j,q) in LUAD patients were associated with poor OS and DFS. High expression of *COL11A1* (Figure 3e), low expression of *FAM107A* (Figure 3f) and *SLIT3* (Figure 3i) were associated with poor OS, but not with DFS.

To elucidate whether these genes were the risk factors for predicting the patients’ survival, we initially performed univariate analysis for the above ten genes. As shown in Table 2, high expression of *CCNB1*,* CENPU*,* GOLM1,* and *TMEM106B* genes was hazard factors for both OS and DFS of LUAD (all of HR >1.36, *p* < 0.05), in the contrary, high expression of *AGER*,* CLIC5,* and *FAM189A2* could promote the OS and DFS of LUAD patients (all of HR <0.73, *p* < 0.05; Table 2).

**Table 2:** Prognosis (OS and DFS) of LUAD patients in a TCGA cohort

	OS				DFS			
	HR (95% CI)[Link]	*p* [Link]	HR (95% CI)[Link]	*p* [Link]	HR (95% CI)[Link]	*p* [Link]	HR (95% CI)[Link]	*p* [Link]
*AGER*	0.640 (0.476–0.860)	0.003	0.558 (0.385–0.810)	0.002	0.627 (0.468–0.839)	0.002	0.514 (0.357–0.740)	0.000
*CCNB1*	1.711 (1.271–2.304)	0.000	1.701 (1.164–2.485)	0.006	1.536 (1.147–2.056)	0.004	1.763 (1.217–2.553)	0.003
*CENPU*	1.549 (1.153–2.081)	0.004	1.631 (1.103–2.414)	0.014	1.360 (1.017–1.818)	0.038	1.541 (1.055–2.252)	0.025
*CLIC5*	0.729 (0.544–0.977)	0.034	0.615 (0.422–0.896)	0.011	0.728 (0.545–0.972)	0.032	0.600 (0.416–0.867)	0.006
*COL11A1*	1.376 (1.024–1.848)	0.034	1.347 (0.930–1.952)	0.115				
*FAM107A*	0.742 (0.553–0.995)	0.046	0.663 (0.458–0.961)	0.030				
*FAM189A2*	0.626 (0.466–0.841)	0.002	0.625 (0.430–0.908)	0.014	0.722 (0.540–0.964)	0.027	0.612 (0.425–0.883)	0.009
*GOLM1*	1.700 (1.267–2.282)	0.000	1.409 (0.977–2.032)	0.067	1.648 (1.232–2.206)	0.001	1.260 (0.881–1.801)	0.206
*SLIT3*	0.635 (0.472–0.854)	0.003	0.628 (0.431–0.915)	0.015				
*TMEM106B*	1.361 (1.014–1.827)	0.040	1.251 (0.869–1.800)	0.228	1.603 (1.195–2.150)	0.002	1.385 (0.968–1.983)	0.075

Notes

CI: confidence interval; DFS: disease‐free survival; HR: hazard ratio; OS: overall survival.

Univariate Cox proportional hazard model.

Multivariate Cox proportional hazard model by adjusting for age (<65 and ≥65), gender (male and female), smoking (<40 and ≥40 pack‐year).

Multivariate proportional hazard models for assessing the association of OS and DFS with these ten genes were subsequently carried out by adjusting age, gender, and smoking pack‐year. It was found that HRs for expression of seven genes (*AGER*,* CCNB1*,* CENPU*,* CLIC5*,* FAM107A*,* FAM189A2,* and *SLIT3*) remained statistically significant for OS and/or DFS after adjusting age, gender, and smoking pack‐year (all of *p* < 0.05, Table 2).

In the following, we analyzed the association of these seven genes expression with OS and/or DFS in LUAD patients at early stages (stage I + II) and advanced stages (stage III + IV) by adjusting age, gender, and smoking pack‐year. As shown in Supporting Information Table S5, *AGER* (HR: 0.598; 95% CI: 0.380–0.841) and *CENPU* (HR: 1.807; 95% CI: 1.108–2.949) expression were associated with OS in patients at early stage, while *SLIT3* (HR: 0.0.439; 95% CI: 0.222–0.869) expression was associated with OS of patients at advanced stage. For DFS, five gene (*AGER*,* CCNB1*,* CENPU*,* COLIL5,* and *FAM189A2*) expressions were found to be associated with DFS in patients at early stage, but none of these genes was related to advanced stage.

Ultimately, using a multivariate Cox regression model, *AGER* was an independent prognostic factor in LUAD for both OS (HR: 0.574; 95% CI: 0.396–0.831) and DFS (HR: 0.617; 95% CI: 0.421–0.905). *CCNB1* was independently associated with DFS for LUAD survival (HR: 1.513; 95% CI: 1.025–2.232; Table 3).

**Table 3:** Multivariate analysis of prognosis of LUAD patients in a TCGA cohort[Link]

Factors	*β*	Standard error	*χ* ^2^	*p*	HR	95% CI for HR	
						Lower	Upper
OS							
*AGER* (high/low)	−0.556	0.189	8.644	0.030	0.574	0.396	0.831
Stage (III + IV/I + II)	0.678	0.240	0.967	0.005	1.970	1.230	3.154
Gender (male/female)	−0.386	0.187	4.267	0.039	0.680	0.472	0.980
DFS							
*AGER* (high/low)	−0.483	0.195	6.109	0.013	0.617	0.421	0.905
*CCNB1* (high/low)	0.414	0.198	4.355	0.037	1.513	1.025	2.232
Stage (III + IV/I + II)	0.670	0.252	7.076	0.008	1.955	1.193	3.204
Age (≥65/<65)	0.516	0.191	7.302	0.007	1.675	1.152	2.435

CI: confidence interval; DFS: disease‐free survival; HR: hazard ratio; OS: overall survival.

Multivariate Cox proportional hazard model by conditional backward method.

## 4 DISCUSSION

With the rapid development of information technology, the ability to collect genomic and clinical information can be used to study disease progression and improve medical treatment (Jiang, Barmada, & Visweswaran, 2010; Schena, Shalon, Davis, & Brown, 1995). One of the growing types of information technology is that obtained from microarray dataset, which was widely used to measure the expression levels of a large number of genes simultaneously.

Oncomine, a cancer microarray database and online data‐mining platform, aimed at promoting discovery from genome‐wide expression analyses (Rhodes et al., 2004). To date, Oncomine contains 715 gene expression datasets and 86,733 samples, in which 74 lung cancer microarray databases are included (https://www.oncomine.org/resource/login.html). There are totally ten datasets containing mRNA expression data of LUAD tissue as well as normal lung tissue. Hereinto, four databases based on microarray platform Human Genome U133 or U133 Plus 2.0 were selected to retrieve mRNA expression information to identify the dysregulated gene in LUAD. As a result, 80 genes significantly dysregulated in LUAD were identified based on microarray database covering 356 cases of LUAD as well as 164 normal lung tissues. Twenty genes were further identified to be consistently dysregulated in all four microarrays by at least twofold. TCGA research network had large numbers of cancer studies and released the databases to the public, including thousands of microarray datasets from lung cancer samples. TCGA has been successfully used to study the association of genes with drug therapy and survival (Shah et al., 2018), endogenous RNA analysis (Ning et al., 2018), and gene–gene interactions (Wu, Huang, & Ma, 2018) in lung cancer. Therefore, in this study, the information of clinical data and mRNA expression in LUAD patients was retrieved from the TCGA database to explore the association of gene expression with survival.

Cancer is considered to be a disease involving dysregulated cell growth, a process in which cells divide uncontrollably. The causes of cancer progression are complex and diverse. Signaling pathways, covering a series of actions among multiple molecules occurring within cells, are important biological mechanisms in cell growth as well as proliferation. Discovering how the pathways and the molecules therein are associated with cancer is one of most essential problems for cancer researchers in the past decades. KEGG is a database resource for understanding high‐level functions and utilities of the biological system, such as the cell, the organism, and the ecosystem, from molecular‐level information, especially large‐scale molecular datasets generated by genome sequencing and other high‐throughput experimental technologies. According to KEGG pathway enrichment analysis by using DAVID, the present study demonstrated that the most significant pathways included the focal adhesion which were closely associated with tumor progression and metastasis. Together, these results indicate that the genes identified in this study might play crucial roles in LUAC progression, probably functioning as a group.

Biomarkers not only have prognostic implications, but are also helpful for measurement of treatment responses and surveillance for tumor recurrence and for guiding clinical decision (Wong, Xu, Chen, Lee, & Luk, 2013). Thus, prognostic biomarkers for LUAD patients are crucial, and there is an ongoing research for predictive biomarkers. Coremine medical mining suggested *AGER* was associated with invasiveness, metastasis, diagnosis, and prognosis and *CCNB1* was associated with invasiveness, metastasis, diagnosis, prognosis, drug resistance, and recurrence.

In this study, a group of genes associated with DFS and OS was identified in 516 LUAD patients. Among these genes, low expression of *AGER*,* CLIC5,* and *FAM189A2*, and high expression of *CCNB1*,* CENPU*,* GOLM1*, and *TEME106B* were associated with poor OS and DFS. High expression of *COL11A1*, low expression of *FAM107A* and *SLIT3* were associated with poor OS, but not with DFS. Furthermore, *AGER* was identified as independent risk prognostic factors for OS and DFS, while *CCNB1* was independently associated with DFS in LUAD patients.

Advanced glycosylation end‐product‐specific receptor (AGER), also named receptor for advanced glycation end products (RAGE) (Ibrahim, Armour, Phipps, & Sukkar, 2013), has been well known as a promoter of inflammation (Nasser et al., 2015). Notably, it has been shown that pulmonary AGER is required for allergen‐induced innate lymphoid cells accumulation in the lung (Oczypok et al., 2015). AGER is one of a limited number of pathogen recognition receptors whose expression is downregulated in lung cancer (Rho, Roehrl, & Wang, 2009; Wang, Li, Yu, et al., 2015). However, AGER has been widely reported being highly expressed in various types of cancer, including ovarian cancer (Rahimi et al., 2017), breast cancer (Nankali et al., 2016), gastric cancer (Wang, Li, Ye, et al., 2015), and endometrial cancer (Zheng et al., 2016). In the current study, we found *AGER* was significantly and consistently downregulated at least 8.266‐fold in LUAD according to four independent microarrays databases. Based on the clinical importance analysis of 516 LUAD patients in a TCGA cohort, low expression of *AGER* was observed to be associated with poor DFS and OS in LUAD patients and was an independent risk prognostic factor for OS. Further study on *AGER* would be needed to better understand its association with LUAD.

CCNB1, an important member of cyclin family, is a key initiator and rigorous quality control step of mitosis. It has a pivotal role in regulating cyclin‐dependent kinase 1 (CDK1) and forming complex with it, which phosphorylates their substrates to promote the transition of cell cycle from G2 phase to mitosis (Krek & Nigg, 1991; Morgan, 1995). Increasing evidence demonstrates that CCNB1 is involved in checkpoint control, whose dysfunction is an early event in tumorigenesis, and that its deregulated expression is observed in a number of different human cancers including breast cancer, cervical cancer, lung cancer, esophageal squamous cell carcinoma, and melanoma (Kedinger et al., 2013; Kreis et al., 2010; Niméus‐Malmström et al., 2010; Nozoe et al., 2002; Yoshida, Tanaka, Mogi, Shitara, & Kuwano, 2004). In parallel, evidence has showed that inhibition of *CCNB1* expression renders breast cancer cells more sensitive to chemotherapy drug taxol (Androic et al., 2008), and CCNB1 is a biomarker for the prognosis of ER + breast cancer and monitoring of hormone therapy efficacy (Ding, Li, Zou, Zou, & Wang, 2014). In addition, CCNB1 is an independent predictor of HBV‐related hepatocellular carcinoma recurrence (Weng et al., 2012). In the present study, our results showed the high expression of *CCNB1* had a poor survival and was an independent factor for the poor DFS in LUAD patients, especially for the patients at the early stage.

## 5 CONCLUSION

In summary, by means of data retrieved from four independent microarrays, clinical importance analyses in a cohort of 516 patients, and bioinformatics analyses including biological process annotation, text mining, we have identified a group of genes that are significantly dysregulated in LUAD and might be associated with cancer progression, development, and in particular, prognosis. *AGER* and *CCNB1* might be useful biomarkers for diagnosis and prognosis and could be potential therapeutic targets for LUAD treatment.

## CONFLICT OF INTEREST

None declared.
