# Supplementary material for: Identification of genes associated with cancer progression and prognosis in lung adenocarcinoma: Analyses based on microarray from Oncomine and The Cancer Genome Atlas databases
Source: Mol Genet Genomic Med. 2018 Dec 16;7(2):e00528. doi: 10.1002/mgg3.528 (PMC6393652; doi:10.1002/mgg3.528)
Supplement: Supplementary file 1 [file MGG3-7-na-s001.docx]

Supplementary table 1. Information of four studies retrieved from the Oncomine database

| Studies | Platform | Normal lung | LUAD |  | Gene measured |
| --- | --- | --- | --- | --- | --- |
| 1. Hou Lung, PLoS One, 2010 [17] | Human Genome U133 Plus 2.0 Array | 65 | 45 |  | 19,574 |
| 1. Landi Lung, PLoS ONE, 2008 [18] | Human Genome U133A Array | 49 | 58 |  | 12,624 |
| 1. Okayama Lung, Cancer Res, 2012 [19] | Human Genome U133 Plus 2.0 Array | 20 | 226 |  | 19,574 |
| 1. Su Lung, BMC Genomics, 2007 [20] | Human Genome U133A Array | 30 | 27 |  | 12,624 |
| Total |  | 164 | 356 |  |  |

Supplementary table 2. Information of genes with stable and consistent dysregulation in four studies

| Gene | Direction of regulation | Full name | Independent microarray data (Fold change) | | | |
| --- | --- | --- | --- | --- | --- | --- |
|  |  |  | Landi Lung | Hou Lung | Su Lung | Okayama Lung |
| *SRPK1* | Up | SRSF protein kinase 1 | 2.03 | 1.831 | 1.716 | 1.849 |
| *HMGB3* | Up | high mobility group box 3 | 4.651 | 5.974 | 3.721 | 4.543 |
| *CCNB1* | Up | cyclin B1 | 2.775 | 6.165 | 3.773 | 2.86 |
| *COL11A1* | Up | collagen type XI alpha 1 chain | 8.356 | 19.936 | 30.562 | 25.236 |
| *GOLM1* | Up | golgi membrane protein 1 | 3.562 | 4.834 | 6.075 | 4.698 |
| *TMEM106B* | Up | transmembrane protein 106B | 2.363 | 2.096 | 2.518 | 2.475 |
| *MLF1IP* | Up | centromere protein U | 2.821 | 3.24 | 2.618 | 2.495 |
| *FAM107A* | Down | family with sequence similarity 107 member A | 10.343 | 6.357 | 6.98 | 6.696 |
| *AGER* | Down | advanced glycosylation end-product specific receptor | 21.35 | 8.266 | 51.087 | 32.945 |
| *GPM6A* | Down | glycoprotein M6A | 7.133 | 11.467 | 9.744 | 13.888 |
| *ADAMTSL3* | Down | ADAMTS like 3 | 3.979 | 4.886 | 5.155 | 5.661 |
| *TGFBR3* | Down | transforming growth factor beta receptor 3 | 5.355 | 6.33 | 4.558 | 4.344 |
| *TNXB* | Down | tenascin XB | 3.14 | 5.207 | 5.245 | 5.734 |
| *ADH1B* | Down | alcohol dehydrogenase 1B (class I), beta polypeptide | 2.929 | 6.436 | 6.863 | 6.893 |
| *CLIC5* | Down | chloride intracellular channel 5 | 8.469 | 7.783 | 4.097 | 5.959 |
| *SLIT3* | Down | slit guidance ligand 3 | 1.776 | 3.51 | 2.974 | 3.371 |
| *SPTBN1* | Down | spectrin beta, non-erythrocytic 1 | 2.248 | 5.392 | 5.204 | 8.158 |
| *EDNRB* | Down | endothelin receptor type B | 8.938 | 6.569 | 5.571 | 7.015 |
| *TNNC1* | Down | troponin C1, slow skeletal and cardiac type | 7.447 | 9.035 | 34.238 | 12.275 |
| *FAM189A2* | Down | family with sequence similarity 189 member A2 | 2.477 | 5.022 | 3.002 | 3.999 |

| Supplementary table3. GO term and KEGG pathway analyses of 80 genes by DAVID online tool   \| Term \| Count \| P Value \| Genes \| \| --- \| --- \| --- \| --- \| \| **Biological Processes** \|  \|  \|  \| \| GO:0030199~collagen fibril organization \| 4 \| 5.94E-04 \| *TNXB, COL1A1, GREM1, COL11A1* \| \| GO:0030198~extracellular matrix organization \| 6 \| 1.44E-03 \| *SPOCK2, PRDX4, COL1A1, COL11A1, SPP1, COL10A1* \| \| GO:0030574~collagen catabolic process \| 4 \| 2.51E-03 \| *COL1A1, COL11A1, MMP12, COL10A1* \| \| GO:0055010~ventricular cardiac muscle tissue morphogenesis \| 3 \| 4.97E-03 \| *TNNC1, TGFBR3, COL11A1* \| \| GO:0045070~positive regulation of viral genome replication \| 3 \| 5.36E-03 \| *ADARB1, TOP2A, SRPK1* \| \| GO:0000281~mitotic cytokinesis \| 3 \| 6.65E-03 \| *NUSAP1, SPTBN1, CEP55* \| \| GO:0001937~negative regulation of endothelial cell proliferation \| 3 \| 6.65E-03 \| *CAV2, CAV1, SULF1* \| \| GO:0042493~response to drug \| 6 \| 9.26E-03 \| *CCNB1, TYMS, SRD5A1, COL1A1, MDK, KCNK3* \| \| GO:0019065~receptor-mediated endocytosis of virus by host cell \| 2 \| 1.26E-02 \| *CAV2, CAV1* \| \| GO:0051726~regulation of cell cycle \| 4 \| 1.56E-02 \| *CCNB1, ADARB1, KIAA0101, GRK5* \| \| GO:0032354~response to follicle-stimulating hormone \| 2 \| 2.10E-02 \| *TGFBR3, SRD5A1* \| \| GO:0070836~caveola assembly \| 2 \| 2.10E-02 \| *CAV2, CAV1* \| \| GO:0007530~sex determination \| 2 \| 2.51E-02 \| *TCF21, SRD5A1* \| \| GO:0051216~cartilage development \| 3 \| 2.58E-02 \| *TYMS, SULF1, COL10A1* \| \| GO:0030512~negative regulation of transforming growth factor beta receptor signaling pathway \| 3 \| 3.00E-02 \| *CAV2, CAV1, TGFBR3* \| \| GO:0001503~ossification \| 3 \| 4.51E-02 \| *IGSF10, COL11A1, SPP1* \| \| **Cell Components** \|  \|  \|  \| \| GO:0005578~proteinaceous extracellular matrix \| 9 \| 1.42E-05 \| *ADAMTS8, TNXB, SPOCK2, ADAMTSL3, TGFBR3, COL11A1, MMP12, COL10A1, SLIT3* \| \| GO:0005581~collagen trimer \| 4 \| 6.47E-03 \| *FCN3, COL1A1, COL11A1, COL10A1* \| \| GO:0005576~extracellular region \| 14 \| 1.36E-02 \| *FGFR4, MDK, AGER, MMP12, SLIT3, IGSF10, FCN3, TGFBR3, SFTPC, ITIH5, COL1A1, COL11A1, SPP1, COL10A1* \| \| GO:0005938~cell cortex \| 4 \| 1.42E-02 \| *CAV1, CLIC5, MELK, COL10A1* \| \| GO:0005794~Golgi apparatus \| 9 \| 2.52E-02 \| *CAV2, KDELR2, FGFR4, CLIC5, SULF1, CA4, COL1A1, GOLM1, AOC3* \| \| GO:0005811~lipid particle \| 3 \| 3.03E-02 \| *CAV1, FABP4, STARD13* \| \| GO:0048471~perinuclear region of cytoplasm \| 7 \| 4.25E-02 \| *CAV2, CAV1, NME1, KIAA0101, CA4, SRD5A1, SPP1* \| \| GO:0005788~endoplasmic reticulum lumen \| 4 \| 4.48E-02 \| *COL1A1, PDIA4, COL11A1, COL10A1* \| \| **Molecular Function** \|  \|  \|  \| \| GO:0008201~heparin binding \| 7 \| 4.83E-05 \| *FGFR4, ADAMTS8, TNXB, TGFBR3, AGER, MDK, SLIT3* \| \| GO:0050840~extracellular matrix binding \| 3 \| 5.02E-03 \| *SPOCK2, COL11A1, SPP1* \| \| GO:0042803~protein homodimerization activity \| 9 \| 9.60E-03 \| *CAV2, TYMS, GGCT, TNNC1, PRDX4, TOP2A, TOX3, KCNK3, AOC3* \| \| GO:0046982~protein heterodimerization activity \| 7 \| 1.17E-02 \| *CAV2, AGTR1, CAV1, PAFAH1B3, TOP2A, KCNK3, AOC3* \| \| GO:0005201~extracellular matrix structural constituent \| 3 \| 3.07E-02 \| *TNXB, COL1A1, COL11A1* \| \| GO:0005113~patched binding \| 2 \| 3.22E-02 \| *CCNB1, CAV1* \| \| GO:0005515~protein binding \| 45 \| 3.27E-02 \| *CAV2, CAV1, FGFR4, HMGB3, MARCKSL1, TNNC1, ADAMTSL3, FHL1, PRDX4, KIAA0101, CEP55, PDIA4, GREM1, STARD13, AGTR1, EDNRB, ASPA, GPM6A, FCN3, PAFAH1B3, SFTPC, TOP2A, GOLM1, SPP1, COL10A1, ADARB1, TNXB, NUSAP1, AGER, TOX3, SRPK1, CCNB1, TMEM106B, PYCR1, NME1, CLIC5, ZWINT, TGFBR3, CA4, SPTBN1, COL1A1, CENPU, GRK5, MELK, AOC3* \| \| GO:0042802~identical protein binding \| 8 \| 3.29E-02 \| *PYCR1, CAV1, CLDN18, NME1, PAFAH1B3, SFTPC, COL1A1, AGER* \| \| GO:0030674~protein binding, bridging \| 3 \| 3.96E-02 \| *CAV2, CAV1, COL11A1* \| \| **KEGG** \|  \|  \|  \| \| hsa04510:Focal adhesion \| 6 \| 3.08E-03 \| *CAV2, CAV1, TNXB, COL1A1, COL11A1, SPP1* \|   Supplementary table 4. The associations of 20 genes with OS and/or DFS of patients with LUAD in a TCGA cohort | | | | | | | | | | | |  |
| --- | --- | --- | --- | --- | --- | --- | --- | --- | --- | --- | --- | --- | --- | --- | --- | --- | --- | --- | --- | --- | --- | --- | --- | --- | --- | --- | --- | --- | --- | --- | --- | --- | --- | --- | --- | --- | --- | --- | --- | --- | --- | --- | --- | --- | --- | --- | --- | --- | --- | --- | --- | --- | --- | --- | --- | --- | --- | --- | --- | --- | --- | --- | --- | --- | --- | --- | --- | --- | --- | --- | --- | --- | --- | --- | --- | --- | --- | --- | --- | --- | --- | --- | --- | --- | --- | --- | --- | --- | --- | --- | --- | --- | --- | --- | --- | --- | --- | --- | --- | --- | --- | --- | --- | --- | --- | --- | --- | --- | --- | --- | --- | --- | --- | --- | --- | --- | --- | --- | --- | --- | --- | --- | --- | --- | --- | --- | --- | --- | --- | --- | --- | --- | --- | --- | --- | --- | --- | --- | --- | --- | --- | --- | --- | --- | --- | --- | --- | --- | --- | --- | --- | --- | --- | --- | --- | --- | --- | --- | --- | --- | --- | --- | --- | --- | --- | --- | --- | --- |
|  | expression | OS | | |  |  | DFS | | |  |  |  |
|  |  | Estimate  median | Median (95% CI) | |  |  | Estimate  median | Median (95% CI) | |  |  |  |
|  |  |  | Lower Bound | Upper Bound | *P* |  |  | Lower Bound | Upper Bound | *P* |  |  |
| *ADAMTSL* | Low | 45.300 | 38.323 | 52.277 | 0.084 |  | 30.980 | 21.870 | 40.090 | 0.656 |  |  |
|  | High | 54.300 | 31.022 | 77.578 |  |  | 41.000 | 29.341 | 52.659 |  |  |  |
| *ADH1B* | Low | 42.480 | 33.153 | 51.807 | 0.054 |  | 27.460 | 22.530 | 32.390 | 0.148 |  |  |
|  | High | 54.300 | 44.847 | 63.753 |  |  | 41.230 | 34.610 | 47.850 |  |  |  |
| *AGER* | Low | 40.370 | 31.335 | 49.405 | **0.003** |  | 26.580 | 23.508 | 29.652 | **0.002** |  |  |
|  | High | 58.800 | 37.020 | 80.580 |  |  | 47.080 | 25.037 | 69.123 |  |  |  |
| *CCNB1* | Low | 54.300 | 46.193 | 62.407 | **0.000** |  | 44.020 | 34.576 | 53.464 | **0.004** |  |  |
|  | High | 42.310 | 31.694 | 52.926 |  |  | 26.580 | 21.614 | 31.546 |  |  |  |
| *CENPU* | Low | 56.670 | 35.126 | 78.214 | **0.003** |  | 41.230 | 32.257 | 50.203 | **0.037** |  |  |
|  | High | 42.480 | 32.924 | 52.036 |  |  | 27.460 | 22.186 | 32.734 |  |  |  |
| *CLIC5* | Low | 44.580 | 35.490 | 53.670 | **0.034** |  | 27.460 | 22.487 | 32.433 | **0.031** |  |  |
|  | High | 53.290 | 43.328 | 63.252 |  |  | 41.000 | 31.008 | 50.992 |  |  |  |
| *COL11A1* | Low | 54.300 | 45.890 | 62.710 | **0.033** |  | 38.370 | 26.814 | 49.926 | 0.182 |  |  |
|  | High | 42.310 | 33.251 | 51.369 |  |  | 33.440 | 21.996 | 44.884 |  |  |  |
| *EDNRB* | Low | 47.770 | 39.888 | 55.652 | 0.083 |  | 30.980 | 21.781 | 40.179 | 0.856 |  |  |
|  | High | 53.610 | 32.496 | 74.724 |  |  | 39.490 | 28.188 | 50.792 |  |  |  |
| *FAM107A* | Low | 47.770 | 39.490 | 56.050 | **0.045** |  | 29.500 | 22.391 | 36.609 | 0.072 |  |  |
|  | High | 53.610 | 33.556 | 73.664 |  |  | 41.330 | 32.273 | 50.387 |  |  |  |
| *FAM189A2* | Low | 39.910 | 31.018 | 48.802 | **0.002** |  | 27.460 | 22.303 | 32.617 | **0.027** |  |  |
|  | High | 58.800 | 33.820 | 83.780 |  |  | 41.330 | 25.559 | 57.101 |  |  |  |
| *GOLM1* | Low | 58.410 | 49.402 | 67.418 | **0.000** |  | 47.540 | 34.101 | 60.979 | **0.001** |  |  |
|  | High | 39.320 | 32.779 | 45.861 |  |  | 26.220 | 21.305 | 31.135 |  |  |  |
| *GPM6A* | Low | 47.770 | 37.555 | 57.985 | 0.342 |  | 27.890 | 24.249 | 31.531 | 0.113 |  |  |
|  | High | 50.200 | 34.872 | 65.528 |  |  | 44.020 | 36.417 | 51.623 |  |  |  |
| *HMGB3* | Low | 45.300 | 39.129 | 51.471 | 0.227 |  | 37.680 | 27.362 | 47.998 | 0.886 |  |  |
|  | High | 54.300 | 25.953 | 82.647 |  |  | 32.330 | 21.297 | 43.363 |  |  |  |
| *SLIT3* | Low | 40.570 | 34.903 | 46.237 | **0.002** |  | 27.890 | 19.376 | 36.404 | 0.135 |  |  |
|  | High | 71.420 | 42.460 | 100.380 |  |  | 41.230 | 30.605 | 51.855 |  |  |  |
| *SPTBN1* | Low | 53.610 | 45.625 | 61.595 | 0.615 |  | 41.330 | 29.662 | 52.998 | 0.177 |  |  |
|  | High | 42.310 | 34.969 | 49.651 |  |  | 29.010 | 19.706 | 38.314 |  |  |  |
| *SRPK1* | Low | 53.610 | 40.747 | 66.473 | 0.379 |  | 37.680 | 28.272 | 47.088 | 0.530 |  |  |
|  | High | 46.680 | 39.424 | 53.936 |  |  | 33.150 | 21.468 | 44.832 |  |  |  |
| *TGFBR3* | Low | 49.310 | 38.453 | 60.167 | 0.780 |  | 27.890 | 19.194 | 36.586 | 0.287 |  |  |
|  | High | 49.240 | 39.148 | 59.332 |  |  | 41.000 | 30.486 | 51.514 |  |  |  |
| *TMEM106B* | Low | 54.300 | 45.054 | 63.546 | **0.039** |  | 47.630 | 24.489 | 70.771 | **0.001** |  |  |
|  | High | 45.300 | 37.451 | 53.149 |  |  | 26.640 | 18.805 | 34.475 |  |  |  |
| *TNNC1* | Low | 47.770 | 39.416 | 56.124 | 0.132 |  | 29.010 | 19.376 | 38.644 | 0.151 |  |  |
|  | High | 50.300 | 39.563 | 61.037 |  |  | 39.490 | 28.368 | 50.612 |  |  |  |
| *TNXB* | Low | 49.210 | 42.342 | 56.078 | 0.291 |  | 27.760 | 20.270 | 35.250 | 0.061 |  |  |
|  | High | 50.200 | 36.234 | 64.166 |  |  | 41.000 | 30.251 | 51.749 |  |  |  |

*P*: Kaplan-Meier survival Log Rank; OS: overall survival; DFS: disease free survival

Supplementary table 5. Prognosis (OS and DFS) of LUAD patients at different stage in a TCGA cohort

|  | OS | | | | |  | DFS | | | | | | | | |  |
| --- | --- | --- | --- | --- | --- | --- | --- | --- | --- | --- | --- | --- | --- | --- | --- | --- |
|  | Stage I+II | |  | Stage III+IV | |  | Stage I+II | | |  | | Stage III+IV | | | |  |
|  | *P* | HR (95% CI) |  | *P* | HR (95% CI) |  | *P* | HR (95% CI) | |  | | *P* | | HR (95% CI) | |  |
| *AGER* | **0.026** | 0.598 (0.380-0.841) |  | 0.058 | 0.531 (0.275-1.023) |  | **0.001** | 0.514 (0.342-0.775) | |  | | 0.054 | | 0.435 (0.186-1.016) | |  |
| *CCNB1* | 0.083 | 1.517 (0.946-2.430) |  | 0.092 | 1.809 (0.908-3.606) |  | **0.018** | 1.657 (1.090-2.519) | |  | | 0.086 | | 2.194 (0.896-5.375) | |  |
| *CENPU* | **0.018** | 1.807 (1.108-2.949) |  | 0.770 | 1.105 (0.564-2.164) |  | **0.007** | 1.810 (1.175-2.787) | |  | | 0.599 | | 0.797 (0.341-1.860) | |  |
| *CLIC5* | 0.125 | 0.699 (0.443-1.104) |  | 0.235 | 0.661 (0.334-1.308) |  | **0.039** | | 0.649 (0.431-0.979) | |  | | 0.082 | | 0.393 (0.137-1.125) | |
| *FAM107A* | 0.210 | 0.747 (0.474-1.178) |  | 0.053 | 0.524 (0.273-1.008) |  |  |  | |  | |  | |  | |  |
| *FAM189A2* | 0.087 | 0.671 (0.425-1.059) |  | 0.582 | 0.825 (0.417-1.633) |  | **0.013** | 0.595 (0.395-0.898) | |  | | 0.765 | | 0.875 (0.364-2.101) | |  |
| *SLIT3* | 0.107 | 0.678 (0.422-1.087) |  | **0.018** | 0.439 (0.222-0.869) |  |  |  | |  | |  | |  | |  |

*multivariate Cox proportional hazard model by adjusting for age (<65 and >=65), gender (male and female), smoking (<40 and >=40 pack-year); OS: overall survival; DFS: disease free survival
